# Identifying Out‐of‐the‐Box Virtual Reality Applications With Geriatric Applicability and Value

**DOI:** 10.1111/jgs.70446

**Published:** 2026-04-16

**Authors:** Alexandra Petrakos, Diana Medved, Amber Winder, Leo L. Nelson, Lexi N. Lindquist, Lee A. Lindquist

**Affiliations:** ^1^ Division of Geriatrics Northwestern University, Feinberg School of Medicine Chicago Illinois USA

**Keywords:** geriatric syndromes, qualitative analysis, virtual reality

## Abstract

**Background:**

Virtual reality (VR) has the potential to improve the quality of life for older adults. Most studies use proprietary VR programs, specifically designed for one goal, which vastly limits the generalizability and use by the older adult population. We sought to identify out of the box (OOTB) VR applications—available to the general population—that have applicability to geriatric care and potential value in improving quality of life.

**Methods:**

We convened and provided VR headsets to an interdisciplinary group of geriatricians, geriatric advanced practice nurses, health services researchers, older adults, and VR advanced users. Panelists were explained the goal of the study and tasked with independently searching and compiling applications that had applicability to geriatrics care or older adults, preferencing those that were cost‐free or low‐cost (< $20). Panel members regularly met to perform constant comparative qualitative analysis in discussions of the identified VR programs. Between meetings, panelists tested the apps that were identified and re‐convened with further information.

**Results:**

Of the over 200 OOTB VR applications examined, panel members identified 44 VR applications that had potential geriatric applicability and value. Potential applications were organized based on price, category, ease of use, and usefulness/applicability to older adults. Panel members reviewed potential negative attributes and applications were ranked/eliminated on perceived usability and utility for older adults. Applications were then thematically analyzed and included (1.) Cognitive support, (2.) Functional Loss/Exercise, (3.) Anti‐anxiety/agitation—relaxation, (4.) Depression alleviating entertainment, and (5.) Isolation‐relieving entertainment.

**Conclusion:**

Out of the box VR experiences can have geriatric applicability and potential value for older adults. Identifying OOTB VR through a consensus panel is the first step to generalizing use in this population. Future research will include testing with older adults, across various settings and levels of physical and cognitive function.

## Summary

- Key points
  ○ The Virtual Reality platform holds promise for interventions to improve the lives of older adults; however, most studies have examined proprietary applications that are only available to those in research studies.
  ○ We convened a panel of geriatric advanced practice nurses (APRNs), geriatricians, geriatrics health services researchers, and VR advanced users who tested over 200 out‐of‐the‐box (OOTB) VR applications and identified 44 which had geriatric applicability and potential value.
  ○ Thematic analysis was used to categorize the applications and geriatric applicability: (1.) Cognitive support (2.) Functional Loss/Exercise), (3.) Anti‐anxiety/agitation—relaxation, (4.) Depression alleviating entertainment (e.g., sports—golfing, fishing, basketball, and (5.) Isolation‐relieving entertainment. (e.g., Pirate battle with others to collect gold, playing tag with a group of gorillas).
  ○ In the future, researchers and older adults may consider using OOTB VR technology to improve geriatric issues such as loneliness, cognitive loss, anxiety, and physical function loss.
- Why does this paper matter?
  ○ No prior studies have examined out‐of‐the‐box (OOTB) VR software specifically for older adult populations. To close this gap, we sought to identify OOTB VR applications—available to the general population—that have applicability to geriatric care and potential value in improving quality of life. We hope that our research stimulates others to pursue VR technology as an intervention to improve the lives of older adults—especially those who are homebound.

## 1 Background

Virtual reality (VR) has recently been shown to have potential to improve the quality of life for older adults [1, 2]. While younger adults are large consumers of VR‐based technology, it has been understudied in its use and ability to support older adults. VR offers an opportunity for older adults who have physical or cognitive limitations to try new experiences (e.g., zipline through picturesque rainforests), visit places from past travel (e.g., Paris, Grand Canyon, Niagara Falls), or lounge on a tropical island beach. Most studies use proprietary VR programs, specifically designed for one goal, which vastly limit the generalizability and use by the older adult population [3, 4]. No prior studies have examined out‐of‐the box (OOTB) VR software specifically for older adult populations. To close this gap, our goal was to identify OOTB VR applications—available to the general population—that have applicability to geriatric care and potential value in improving quality of life.

## 2 Methods

We convened and provided the Oculus Quest 2 VR headset to an interdisciplinary group of geriatric advanced practice nurses (*n* = 2, mean years of experience: 7.5 years), geriatricians (*n* = 2, mean years of experience: 11.0 years), geriatrics health services researchers (*n* = 2), older adults (*n* = 4, mean age: 79.0 years), and VR advanced users (*n* = 4, mean age: 23.0 years). Group members were volunteers recruited from networks connected to the Northwestern University Division of Geriatrics and had to be able to use the internet and learn how to use the Oculus VR headset. They were explained the goal of the project and independently searched and compiled applications that had applicability to geriatrics care or older adults, preferencing those that were cost‐free or low‐cost (< $20). After generating the lists, panelists ranked the ease of use from 1 to 10, with 1 being easiest for older adults, positives/negatives/ and potential themes for geriatric applicability. Led by the geriatrician principal investigator (AP), panel members regularly met monthly in small groups to perform constant comparative qualitative analysis in discussions of the identified VR programs. Between meetings, panel members re‐tested the apps that were identified and then reconvened with further information.

## 3 Results

**FIGURE 1:**
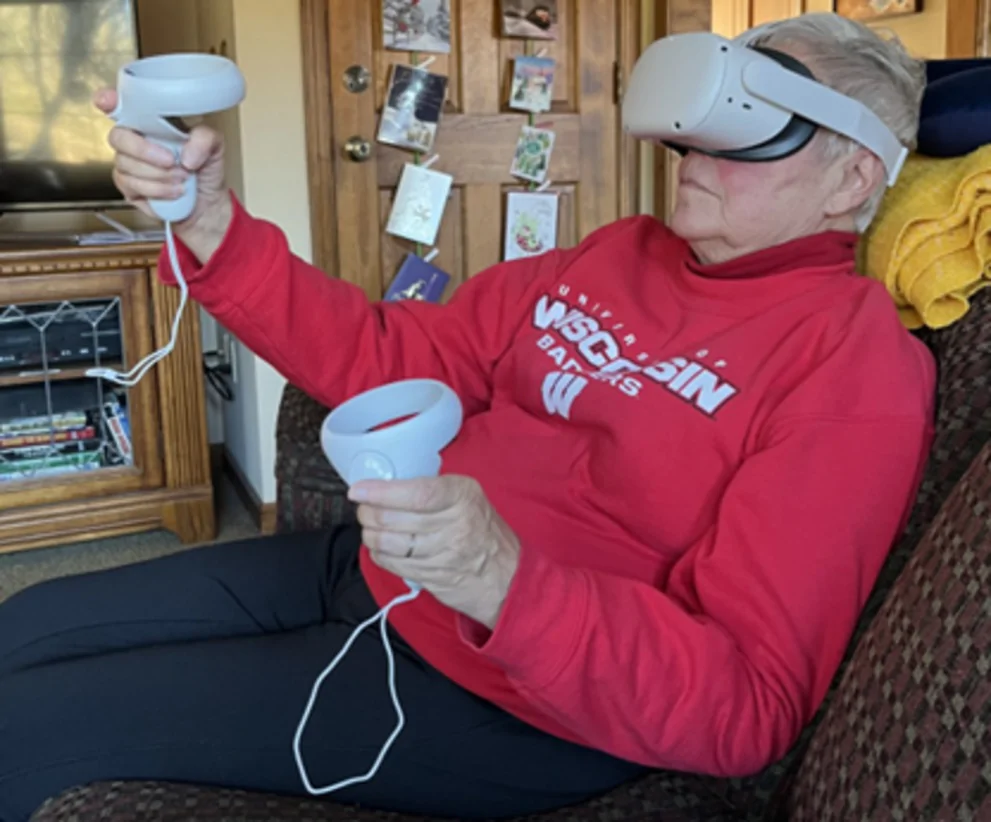
Panelist using the Oculus VR Technology.

Of the over 200 OOTB VR applications examined, panel members identified 44 VR applications that had potential geriatric applicability and value, between January 2024 and May 2025 (Figure 1). Potential applications were organized based on price, category, ease of use, and usefulness/applicability to older adults. Panel members reviewed potential negative attributes of each application and applications were ranked/eliminated on perceived usability and utility for older adults. Negative attributes included realistic experiences that may be traumatizing to older adults (e.g., realistic shooting during a war, burial sequences, angel of death appearances). For perceived usability, panelists considered ease of learning to use the application from start‐up, geriatric traits (e.g., low vision, reaction times, muscular‐skeletal abilities) and “choke points” where the application had parts that were difficult to move past causing frustration. Consensus was reached on 44 applications and 13 were selected as high quality for older adults. These were deemed easy to use, had geriatric applicability and potential value to older adults. Applications were then thematically analyzed with the resulting geriatric applicability (1.) Cognitive support (e.g., pattern memorization, interactive puzzles), (2.) Functional Loss/Exercise (e.g., punching musical beats during a song; using a handcar to go through caves, completing tai chi motions), (3.) Anti‐anxiety/agitation—relaxation (e.g., calming music with visual patterns, participating in meditation techniques, looking around a calm seashore), (4.) Depression alleviating entertainment (e.g., sports—golfing, fishing, basketball, skiing; gambling—playing poker, craps, roulette, music—attending a rock concert), and (5.) Isolation‐relieving entertainment. (e.g., Pirate battle with others to collect gold, playing tag with a group of gorillas). Several of the applications touched on more than one thematic area which panelists felt would increase applicability (Table 1). Included in Table 2 are the 44 OOTB VR applications with green denoting the highest applicability and potential for older adults.

**TABLE 1:** Geriatric applicability of virtual reality applications.

Geriatric applicability (themes)	Example apps
Memory Loss (Cognition Support)	Memorizing patterns Interactive 3D puzzles Looking through locations
Functional Loss (Exercise)	Hitting beats with saber Completing tai chi motions Hand pump train car
Anxiety and Agitation (Relaxation)	Walking through nature, Meditative breathing
Depression (Entertainment)	Playing golf, Tennis, Simulated Fishing
Isolation (Action/Adventure, Entertainment)	Pirate battle to collect gold, Ziplining through forest, Playing tag with gorillas

**TABLE 2:** Virtual reality applications reviewed by panel for older adult applicability.

App/Game Name	Description	Ease of Startup/Use 1 (easy)—10 (hard)	Geriatric Usage	Positives	Negatives	User Notes
Bait	Go fishing in a fantasy world with various calming locations and types of fish to catch	1	Physical Function, Cognitive Stim, Relaxation	Relaxing, Can do while sitting, social—can fish with others	Tiring for arms, Frustrating if not catching fish	Easy to administer. Good for stationary patients; need to maintain grip for long periods.
Holofit	Go on scenic journeys while doing cardio exercises to keep progressing.	4	Physical Function, Cognitive Stim.	Pretty scenery Great exercise More than 100 workouts	Disorienting and darkness at points, not for fear of heights.	Stationary/active options. Cycling, skiing, kayaking. Easy to use when started.
Kizuna Al—Touch the Beat	Hit oncoming notes with glowsticks to the beat of songs.	1	Physical Function, Cognitive Stim.	Active, Fun dancer, Free Alternative to others	Tiring for arms, May not be able to hit all beats, High Contrast	Small variety of music, but fun to dance. Accessible for older adults
Gorilla Tag	Become a legless gorilla and use hands to maneuver obstacles and play tag with others.	4	Physical Function, Cognitive Stim., Social	Active and can connect with friends. Unique perspectives.	Multiplayer with other people but able to mute them	Panel Favorite. Liked by everyone
Cosmic Flow	Enter a meditative experience designed to relax and calm.	1	Relaxation Anxiety	Relaxing. Can sit or lay down to do	None identified	Panel really liked. Can also stand and feel like floating. Easy to administer.
Puzzling places	Complete 3D jigsaw puzzles of realistic miniature landmarks	1	Cognitive Stim, Memory	Good for Cognition	Doing puzzles	Engaging. Can be stationary. Potential for cognition.
National Geographic	Explore locations, encounter sights and animals.	1	Physical Function, Cognitive Stim.	Active, exploring and adventure	None identified	Easy to administer, panel really liked. Can take pictures for more engagement.
YouTube	Watch immersive VR videos uploaded by the community.	1	Cognitive Stim., Relaxation Memory	Good to revisit places, Plenty of options like ziplining	None identified	Selecting right videos takes some effort, large number of places exist
Epic Roller Coasters	Ride imaginative coasters through fantasy worlds, playing games.	1	Cognitive Stim., Memory	Relaxing but interactive	Not for people who get easily dizzy	Tutorial was fun, need to pick a specific game or too much to guide on how to start.
TRIPP	Engage in fantasy environments to improve wellbeing.	1	Relaxation Anxiety, Mood	Good for mood, engage, Mindfulness	None identified	Super easy, Similar to cosmic flow. Potential for anxiety.
Match Point Tennis	Play tennis in various locations with others or AI opponents.	2	Physical Function	Simulation Tennis	None identified	easy, doesn't require much grip strength, can sit
Golf+	Play golf in official courses that follow the PGA Tour.	2	Physical Function	Stimulating Golf especially for users	None identified	Seems easy to do. Fun to do and stimulating.
Beat Saber	Hit oncoming beats with glowsticks to the song rhythm.	2	Physical Function, Cognitive Stim, Memory	Great music	Depends on the user's music preferences	Could be stationary and move arms or be active/standing.
Star Chart	Explore space, visualize constellations.	2		Exploration, education, relaxing	None identified	Would be easy to administer. Also can be done stationary.
Rec Room	Play variety of community‐made games with option to socialize with others.	2		Wide variety games. (Bowling, Laser Tag.)	Unclear interactions in games.	More specific games listed to minimize need for long tutorial
Echo VR	Become a robot and play a zero‐gravity version of frisbee.	3		Zero G so it feels like you can move free	Hard to learn. Need to recall use of controls.	Tutorial is long, May be too intense.*
Population: ONE	Play against others and use weapons to win a battle royale.	2		Fun tutorial	A lot of arm motions for climbing	Accessing the game is difficult, In public space—kids playing.
Ultimechs	Play soccer as a robot.	2		None identified	Not ideal for older adults	Good if want to be a transformer.
Litesport	Exercise in added VR environments.	4		None identified	Hard to use	Different work outs, more intensive
Sail	Sail and hunt for treasure as a pirate.	3		None identified	Too difficult	Difficult moving to places.
Sports Scramble	Play sports in fun environments.	2		None identified	Need to pay to play	Too many add‐ons
Poker VR	Play poker with others.	3		Seems simple	Need to pay to play	Arm movements need to learn
Vegas infinite	Interactive casino, playing games in fantasy worlds.	3		None identified	Difficult to navigate and control cards.	Easiest to use is the slot machine which was rather boring.
Supernatural	Exercise in high‐intensity other worldly locations.	3		None identified	Complicated, Requires Subscription	Model like bike programs. costly
Open Brush	Create immersive art pieces with special VR effects.	6		None identified	Difficult to navigate	Hard to get to canvas to actually paint.
I Expect You To Die	Progress through story‐based escape rooms as a spy.	6		Creatively think with objects.	Intense—Death, Smoke, Flashing lights.	May be too much pressure. Jump scares and explosions.
Gravity Sketch	Create sketches in VR	3		Mainly coloring	Boring, not social	Too complicated
Goliath: Playing with Reality	Enter an immersive story with abstract settings.	7		None identified	Very dark, could not see far away, flashing lights	Lack of contrast not ideal for older adults
Gun Raiders	Play shooting games with other players.	7		None identified	Difficult to navigate	Not ideal for older adults, hard to track.
Journey of the Gods	Explore a unique fantasy world	7		None identified	It was very dark and hard to see.	Seems too serious
MeetinVR	3D workspaces for remote collaboration	7		Socialization is possible.	Requires business account	Not useful for seniors, not entertaining.
Superhot	Eliminate enemies in slow‐motion puzzles as time moves when you move.	3		Active; works on balance (freezing in positions)	Intense—Jump scares, Fights, Guns, Smoke after death	High contrast; Very intense and challenging for older adults.
The Under Presents	Progress through fantasy stories.	3		None identified	Difficult to understand	Unclear what the point is.
Venues by Facebook	Talk to real people, make friends in a tranquil park.	3		Social, Special seasonal animation	Not active, difficult if you're not social	Learn how to take selfies. Becomes boring after a while.
Wander	Explore different eras in time while solving puzzles.	3		Able to return to eras from childhood.	None identified	Panel preferred national geographic
Moonrider	Punch beats to the rhythm of a large variety of songs.	5		None identified	Hard to use	Difficult to understand for older adults
Google Earth	Explore the earth and locales of your choosing.	5		Able to travel around earth	None identified	Panel liked Map better.
Smash Drums	Smash targets on drums to the beat.	5		None identified	Challenging start‐up	Not ideal for older adults
X8	Eliminate others with unique abilities.	5		None identified	Violent at times	Not enjoyable to panel members
Big Ballers vr	Play sports using real world game rules.	3		None identified	Needs strength	Tailored toward young population
Cards and Tankards	Play card games at a social tavern.	6		None identified	Hard to understand	Not ideal for older adults
Wooorld	Explore the globe—interact with others.	5		Social, easy to use	Hand moving to change position.	Panel preferred national geographic
Elixir	Create potions as a magical apprentice.	6		Use hands not controller	Not practical to play sitting.	Setup is too complicated.
Immerse	Learn languages in real environments.	6		Offers four languages	Difficult to get through	Lukewarm. Requires membership.

*Note:* Green indicates the highest applicability potential for older adults, while Red denotes the lowest applicability potential.

* No longer active.

## 4 Conclusion

Out of the box VR experiences can have geriatric applicability and potential value to older adults. Identifying OOTB VR through a consensus panel is the first step to generalizing use in this population. With the large number of available OOTB applications, older adults may benefit from a curated collection to augment their preferences and tailor their individual VR use. As VR technology grows, more OOTB applications are becoming available and older versions are evolving. One limitation of this study is the timeline as even more applications are available now than when our panel initially convened. Prior to our final submission, we reviewed the apps to ensure that they were active and only one was found discontinued. Hopefully in the future, more OOTB VR applications will be targeted to improve the lives of older adults. While older adults participated in the panel, future large cohort research is needed to test the VR applications with older adults, across settings (e.g., independent, homebound, assisted living, nursing home) and physical and cognitive function levels.

## Author Contributions

All authors met criteria for authorship by (1) providing substantial intellectual contribution to the study's conception and design (A.P., D.M., A.W., L.A.L.), data acquisition (all authors), data analysis (A.P., D.M., A.W., L.A.L.), and interpretation (A.P., D.M., A.W., L.A.L.); drafting the article or revising it critically for important intellectual content (all authors); and approving the final version to be published (all authors).

## Funding

This research is supported through grants from the NIH/NIA R01AG068421, R01AG058777, R01AG083034, and P30AG059988.

## Disclosure

The sponsor was not involved in the design, methods, analysis, and interpretation of the data, and preparation of the manuscript.

## Conflicts of Interest

The authors declare no conflicts of interest.
